# A Protein Turnover Signaling Motif Controls the Stimulus-Sensitivity of Stress Response Pathways

**DOI:** 10.1371/journal.pcbi.1002932

**Published:** 2013-02-28

**Authors:** Paul Michael Loriaux, Alexander Hoffmann

**Affiliations:** 1 Signaling Systems Laboratory, Department of Chemistry and Biochemistry, University of California San Diego, La Jolla, California, United States of America; 2 Graduate Program in Bioinformatics and Systems Biology, University of California San Diego, La Jolla, California, United States of America; 3 The San Diego Center for Systems Biology, La Jolla, California, United States of America; North Carolina State University, United States of America

## Abstract

Stimulus-induced perturbations from the steady state are a hallmark of signal transduction. In some signaling modules, the steady state is characterized by rapid synthesis and degradation of signaling proteins. Conspicuous among these are the p53 tumor suppressor, its negative regulator Mdm2, and the negative feedback regulator of NFκB, IκBα. We investigated the physiological importance of this turnover, or flux, using a computational method that allows flux to be systematically altered independently of the steady state protein abundances. Applying our method to a prototypical signaling module, we show that flux can precisely control the dynamic response to perturbation. Next, we applied our method to experimentally validated models of p53 and NFκB signaling. We find that high p53 flux is required for oscillations in response to a saturating dose of ionizing radiation (IR). In contrast, high flux of Mdm2 is not required for oscillations but preserves p53 sensitivity to sub-saturating doses of IR. In the NFκB system, degradation of NFκB-bound IκB by the IκB kinase (IKK) is required for activation in response to TNF, while high IKK-independent degradation prevents spurious activation in response to metabolic stress or low doses of TNF. Our work identifies flux pairs with opposing functional effects as a signaling motif that controls the stimulus-sensitivity of the p53 and NFκB stress-response pathways, and may constitute a general design principle in signaling pathways.

## Author Summary

Eukaryotic cells constantly synthesize new proteins and degrade old ones. While most proteins are degraded within 24 hours of being synthesized, some proteins are short-lived and exist for only minutes. Using mathematical models, we asked how rapid turnover, or *flux*, of signaling proteins might regulate the activation of two well-known transcription factors, p53 and NFκB. p53 is a cell cycle regulator that is activated in response to DNA damage, for example, due to ionizing radiation. NFκB is a regulator of immunity and responds to inflammatory signals like the macrophage-secreted cytokine, TNF. Both p53 and NFκB are controlled by at least one flux whose effect on activation is positive and one whose effect is negative. For p53 these are the turnover of p53 and Mdm2, respectively. For NFκB they are the TNF-dependent and -independent turnover of the NFκB inhibitor, IκB. We find that juxtaposition of a positive and negative flux allows for precise tuning of the sensitivity of these transcription factors to different environmental signals. Our results therefore suggest that rapid synthesis and degradation of signaling proteins, though energetically wasteful, may be a common mechanism by which eukaryotic cells regulate their sensitivity to environmental stimuli.

## Introduction

Eukaryotic cells must constantly recycle their proteomes. Of the approximately 10^9^ proteins in a typical mouse L929 fibrosarcoma cell, 10^6^ are degraded every minute [1]. Assuming first-order degradation kinetics, this rate of constitutive protein turnover, or *flux*, imposes an average half-life of 24 hours. Not all proteins are equally stable, however. Genome-wide quantifications of protein turnover in HeLa cells [2], [3] and 3T3 murine fibroblasts [4] show that protein half-lives can span several orders of magnitude. Thus while some proteins exist for months and even years [5], others are degraded within minutes. Gene ontology terms describing signaling functions are highly enriched among short-lived proteins [3], [6], [7], suggesting that rapid turnover is required for proper signal transduction. Indeed, defects in protein turnover are implicated in the pathogenesis of cancer and other types of human disease [8], [9].

Conspicuous among short-lived signaling proteins are those that regulate the p53 and NFκB stress response pathways. The p53 protein itself, for example, has a half-life of less than 30 minutes [10], [11]. Mdm2, the E3 ubiquitin ligase responsible for regulating p53, has a half-life of 45 minutes [4]. And the half-life of unbound IκBα, the negative feedback regulator of NFκB, is less than 15 minutes [12], [13] (see Figure S1), requiring that 6,500 new copies of IκBα be synthesized every minute [13]. Given the energetic costs of protein synthesis, we hypothesized that rapid turnover of these proteins is critical to the stimulus-response behavior of their associated pathways.

To test our hypothesis we developed a method to systematically alter the rates of protein turnover in mass action models without affecting their steady state abundances. Our method requires an analytical expression for the steady state of a model, which we derive using the *py*-substitution method described in a companion manuscript. From this expression, changes in parameter values that do not affect the steady state are found in the null space of the matrix whose elements are the partial derivatives of the species abundances with respect to the parameters. We call this vector space the *isostatic subspace*. After deriving a basis for this subspace, linear combinations of basis vectors identify *isostatic perturbations* that modify specific reactions independently of all the others, for example those that control protein turnover. By systematic application of these isostatic perturbations to a model operating at steady state, the effects of flux on stimulus-responsiveness can be studied in isolation of changes to steady-state abundances (see Methods).

We first apply our method to a prototypical negative feedback module in which an activator controls the expression of its own negative regulator. We show that reducing the flux of either the activator or its inhibitor slows the response to stimulation. However, reducing the flux of the activator lowers the magnitude of the response, whereas reducing the flux of the inhibitor increases it. This complementarity allows the activator and inhibitor fluxes to exert precise control over the module's response to stimulation.

Given this level of control, we hypothesized that rapid turnover of p53 and Mdm2 must be required for p53 signaling. A hallmark of p53 is that it responds to DNA damage in a series of digital pulses [14]–[18]. These pulses are important for determining cell fate [19]–[21]. To test whether high p53 and Mdm2 flux are required for p53 pulses, we applied our method to a model in which exposure to ionizing radiation (IR) results in oscillations of active p53 [17]. By varying each flux over three orders of magnitude, we show that high p53 flux is indeed required for oscillations. In contrast, high Mdm2 flux is not required, but rather controls the refractory time in response to transient stimulation. If the flux of Mdm2 is low, a second stimulus after 22 hours does not result in appreciable activation of p53.

In contrast to p53, the flux of NFκB turnover is very low, while the flux of its inhibitor, IκB, is very high. Prior to stimulation, most NFκB is sequestered in the cytoplasm by IκB. Upon stimulation by an inflammatory signal like tumor necrosis factor alpha (TNF), IκB is phosphorylated and degraded, resulting in rapid but transient translocation of NFκB to the nucleus and activation of its target genes [22]–[24]. Two separate pathways are responsible for the turnover of IκB [12]. In one, IκB bound to NFκB is phosphorylated by the IκB kinase (IKK) and targeted for degradation by the ubiquitin-proteasome system. In the other pathway, unbound IκB is targeted for degradation and requires neither IKK nor ubiquitination [25], [26]. We call these the “productive” and “futile” fluxes, respectively. Applying our method to a model of NFκB activation, we show that the futile flux acts as a negative regulator of NFκB activation while the productive flux acts as a positive regulator. We find that turnover of bound IκB is required for NFκB activation in response to TNF, while high turnover of unbound IκB prevents spurious activation of NFκB in response to low doses of TNF or ribotoxic stress caused by ultraviolet light (UV). As with p53 then, juxtaposition of a positive and negative regulatory flux govern the sensitivity of NFκB to different stimuli, and may constitute a common signaling motif for controlling stimulus-specificity in diverse signaling pathways.

## Results

### Activator and inhibitor fluxes can precisely control the dynamics of signaling

**Figure 1:**
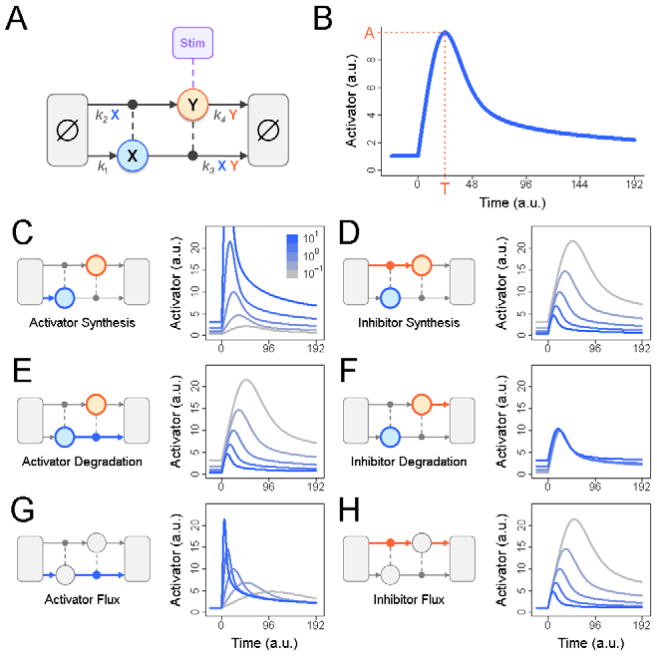
A prototypical negative feedback module. (A) In this simple model of negative feedback control, an activator X is constitutively produced but catalytically degraded by an inhibitor, Y. Y is constitutively degraded but its synthesis requires X. Each of these four reactions is modeled using mass action kinetics. To stimulate the model and activate X, the steady-state abundance of Y is instantaneously depleted. (B) In response to stimulation, the abundance of X increases until activator-induced synthesis of Y forces a return to steady-state. This response can be characterized by 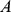, the maximum abundance of X following stimulation, and 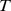, the time at which 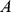 is observed. Parameters were chosen for this model such that the steady state abundances of X and Y equal one arbitrary unit and the stimulus-induced amplitude of X is 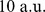 at time 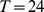. The rates of activator synthesis (C), inhibitor synthesis (D), activator degradation (E), and inhibitor degradation (F) were multiplied by 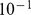 (gray) to 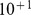 (blue) prior to stimulation as described above. For each multiplier, the dynamics of the activator response are plotted on the right. Similar plots were generated by multiplying the flux of the activator (G), and the flux of the inhibitor (H), as described in Methods.

To examine the effects of flux on stimulus-responsiveness, we built a prototypical negative feedback model reminiscent of the p53 or NFκB stress-response pathways (Figure 1A). In it, an activator “X” is constitutively expressed but catalytically degraded by an inhibitor, “Y”. The inhibitor is constitutively degraded but its synthesis requires X. Activation is achieved by instantaneous depletion of Y, the result of which is accumulation of X until negative feedback forces a return to steady state. The dynamics of this response can be described by two values: 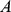, the amplitude or maximum value of X after stimulation, and 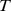, the time at which 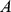 is observed (Figure 1B). Parameters for this model were chosen such that the abundances of both X and Y are one arbitrary unit and X achieves its maximum value of 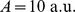 at time 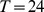, where the units of time are also arbitrary.

To address the role of these parameters in shaping the response of the activator, we first performed a traditional sensitivity analysis. We found that increasing the synthesis of X (Figure 1C), or decreasing the degradation of X (Figure 1D) or the synthesis of Y (Figure 1E), all result in increased responsiveness. However, these changes also increase the abundance of X. To distinguish between the effects caused by changes in flux and those caused by changes in abundance, we developed a method that alters the flux of X and Y while maintaining their steady state abundances at 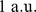. Using this method, we found that increasing the flux of X increases responsiveness (Figure 1G), but not to the same extent as increasing the synthesis parameter alone (Figure 1C). In contrast, reducing the flux of Y yields the same increase in responsiveness as decreasing the synthesis of Y (Figure 1E) or the degradation of X (Figure 1D). These observations suggest that both the flux and abundance of X are important regulators of the response, as is the flux of Y, but not its abundance. This conclusion is supported by the observation that when the abundance of Y is increased by reducing its degradation, there is little effect on signaling (Figure 1F).

**Figure 2:**
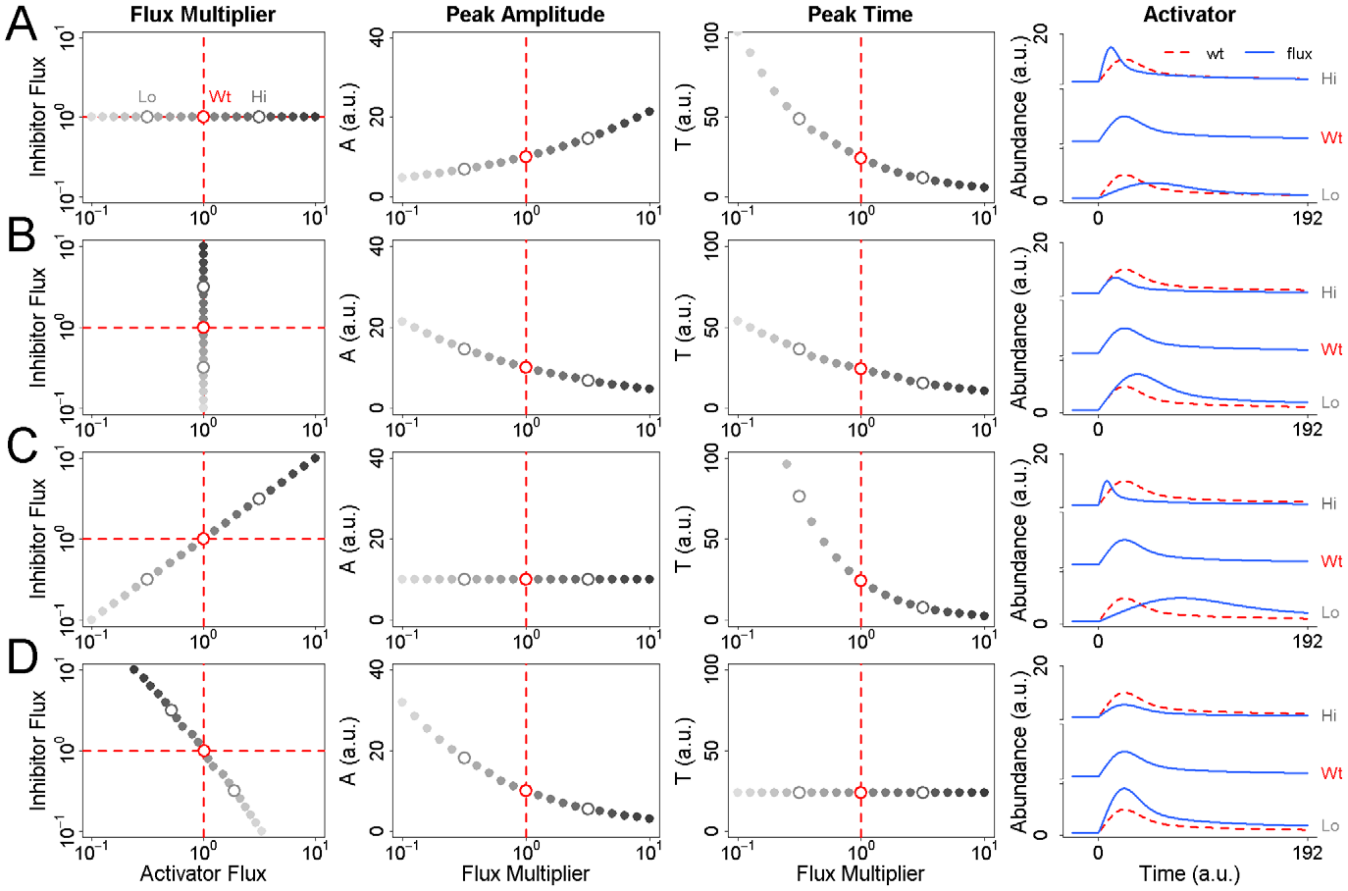
Effects of flux on the dynamic response to stimulation. (A) The magnitude of the activator flux is varied between 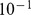 (light gray) and 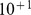 (dark gray) times its nominal steady-state value prior to stimulation. The peak amplitude 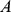 of X in response to stimulation is observed to increase with the flux of X while the time 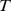 at which the peak occurs is observed to decrease. Representative profiles of the activator at low, wildtype, and high values of the flux are shown at right. The dashed red line indicates the nominal wildtype response. (B) The magnitude of the inhibitor flux is varied between 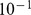 and 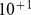 times its nominal steady-state value prior to stimulation. Both 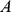 and 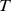 are observed to decrease. (C) The fluxes of both X and Y are varied simultaneously between 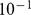 and 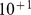 times their nominal wildtype values. As a result, 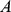 is held constant while 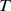 is reduced. (D) The magnitude of the inhibitor flux is varied between 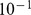 and 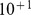 times its nominal steady-state value prior to stimulation. For each value of this flux, the value of activator flux is calculated such that 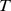 is held constant. As in row 2 above, 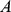 is observed to decrease as the magnitude of the flux of Y increases.

To further characterize the effects of flux on the activator's response to stimulation, we applied systematic changes to the fluxes of X and Y prior to stimulation and plotted the resulting values of 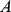 and 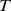. Multiplying the flux of X over the interval 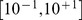 showed, as expected, that the value of 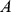 increases while the value of 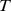 deceases (Figure 2A). In other words, a high activator flux results in a strong, fast response to stimulation. If we repeat the process with the inhibitor, we find that both 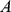 and 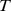 decrease as the flux increases; a high inhibitor flux results in a fast but weak response (Figure 2B). This result illustrates that fluxes of different regulators can have different but complementary effects on stimulus-induced signaling dynamics.

Complementarity suggests that changes in flux can be identified such that 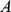 is altered independently of 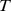, or 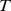 independently of 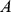. Indeed, if both activator and inhibitor fluxes are increased in equal measure, 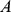 is held fixed while the value of 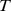 decreases (Figure 2C). Increasing both fluxes thus simultaneously reduces the timescale of the response without affecting its magnitude. An equivalent relationship can be found such that 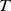 remains fixed while 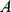 is affected (Figure 2D). Because an increase in either flux will reduce 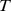, to alter 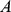 without affecting 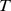 requires an increase in one flux but a decrease in the other. Also, 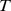 is more sensitive to changes in the inhibitor flux *versus* the activator flux; small changes in the former must be paired with larger changes in the latter. This capability to achieve any value of 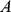 or 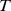 indicates that flux can precisely control the response to stimulation, without requiring any changes to steady state protein abundance.

### High p53 and Mdm2 flux is required for p53 responsiveness to ionizing radiation

**Figure 3:**
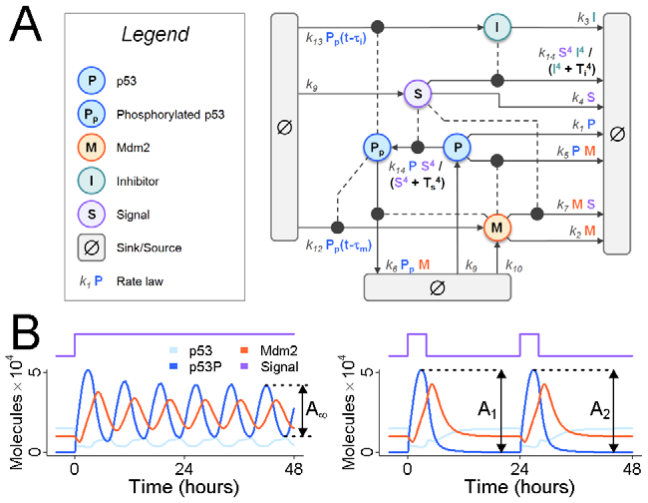
A model of p53 oscillations in response to ionizing radiation. (A) The model shown here is structurally identical to [17], but parameter values have been scaled to match published rates of synthesis and degradation for p53 and Mdm2 as well as their steady-state abundances (see Methods). (B) Ionizing radiation is modeled as an increase in synthesis of the Signal molecule (left; model parameter 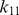) [17]. In response to a step increase in Signal production, phosphorylated p53 is observed to oscillate. We define 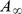 to be the amplitude of the stable oscillations. In response to a 2-hour pulse in Signal production (right), p53 exhibits a transient peak in phosphorylation, as does Mdm2. We define 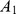 to be the amplitude of phosphorylated p53, and 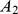 to be its amplitude in response to a second, identical pulse, 22 hours after the first pulse.

Given that flux precisely controls the dynamic response to stimulation in a prototypical signaling module, we hypothesized that for p53, oscillations in response to DNA damage require the high rates of turnover reported for p53 and Mdm2. To test this, we applied our method to a published model of p53 activation in response to ionizing gamma radiation (IR), a common DNA damaging agent (Figure 3A) [17]. Because the model uses arbitrary units, we rescaled it so that the steady state abundances of p53 and Mdm2, as well as their rates of synthesis and degradation, matched published values (see Table S1). We note that these values are also in good agreement with the consensus parameters reported in [16].

**Figure 4:**
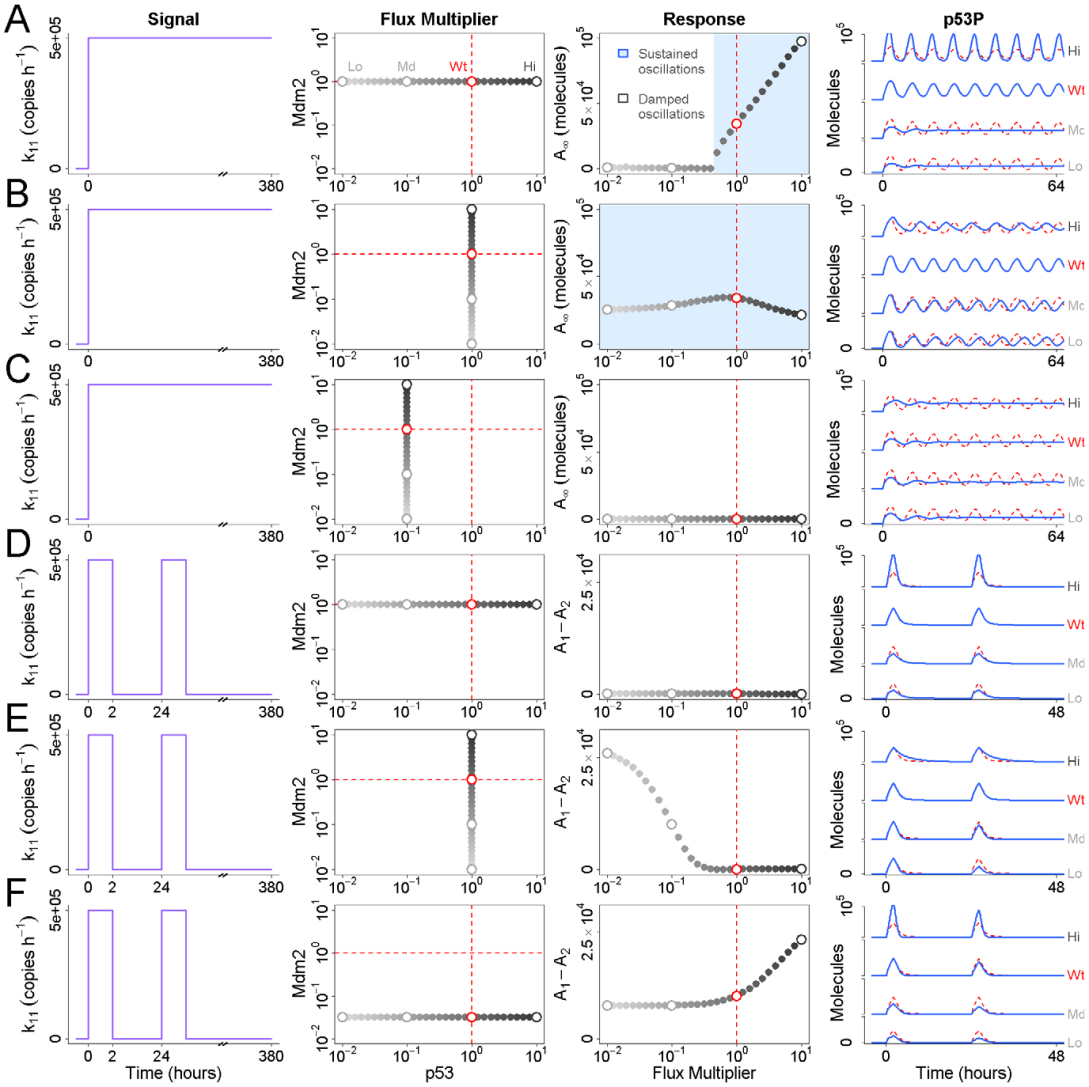
Effects of flux on the p53 response to ionizing radiation. (A) The Mdm2-independent flux of p53 is varied between 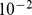 and 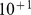 times its wildtype value prior to a step increase in Signal production (model parameter 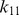; light gray to dark gray) [17]. The magnitude of 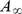 is plotted as a function of this p53 flux multiplier. Values that give rise to stable oscillations are shaded in blue. At right, representative profiles of phosphorylated p53 are shown for high, wildtype, moderate, and low values of the multiplier. Note that the wildtype flux is indicated by the dashed line in red. (B) As row 1, above, but now the p53-independent flux of Mdm2 is varied between 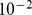 and 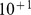 times its wildtype value (light gray to dark gray). Stable oscillations are observed for all values of the Mdm2 flux multiplier. (C) As row 2, above, but for all simulations the flux of p53 is at one-tenth its nominal wildtype value. Instead of sustained oscillations, damped oscillations are observed for all values of the Mdm2 flux multiplier. (D) The flux of p53 is varied between 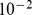 and 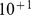 times its wildtype value prior to a 2-hour pulse in Signal production, followed by 22 hours of rest, followed by a second 2-hour pulse. No difference is observed in the amplitude of phosphorylated p53 in response to the first and second pulse. (E) As row 4, above, but now the flux of Mdm2 is varied instead of p53. At lower values of the Mdm2 flux multiplier, a significant difference is observed between the amplitude of phosphorylated p53 in response to the first and second pulse. (F) As row 4, above, but while the p53 flux is allowed to vary, the flux of Mdm2 is held constant at 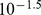 times its wildtype value. This concomitant reduction of the p53 flux is not able to rescue the Mdm2-compromised response to the second pulse.

Next we implemented a multiplier of Mdm2-independent p53 flux and let it take values on the interval 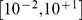. For each value we simulated the response to IR using a step function in the production of the upstream Signal molecule, 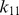, as previously described [17]. To characterize the p53 response we let 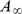 be the amplitude of stable oscillations in phosphorylated p53 (Figure 3B), and use this as a metric for p53 sensitivity. Where 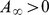, we say the module is sensitive to IR stimulation. We find that 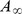 is greater than zero only when the flux of p53 is near its observed value or higher (Figure 4A). If the flux of p53 is reduced by 2-fold or more, p53 no longer stably oscillates in response to stimulation, but exhibits damped oscillations instead.

Interestingly, repeating this analysis with a multiplier for the Mdm2 flux over the same interval reveals that Mdm2 flux has little bearing on p53 oscillations (Figure 4B). For any value of the multiplier chosen, 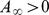. As with p53, this multiplier alters the Signal-independent flux of Mdm2 but does not affect Signal-induced Mdm2 degradation. If oscillations are already compromised by a reduced p53 flux, no concomitant reduction in Mdm2 flux can rescue the oscillations (Figure 4C). We therefore conclude that the flux of p53, but not Mdm2, is required for IR-sensitivity in the p53 signaling module. What then is the physiological relevance of high Mdm2 flux? In the model, signal-mediated Mdm2 auto-ubiquitination [27] is a major contributor to Mdm2 degradation after stimulation. If Signal production is transient, Mdm2 protein levels must be restored solely via Signal-independent degradation. We therefore hypothesized that if the flux of Mdm2 is low, Mdm2 protein levels would remain elevated after stimulation and compromise sensitivity to subsequent stimuli.

To test this hypothesis we again let the Mdm2 flux multiplier take values over the interval 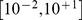. For each value we stimulated the model with a 2-hour pulse of Signal production, followed by 22 hours of rest, followed by a second 2-hour pulse (Figure 3B). We defined 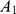 to be the amplitude of the first peak of phosphorylated p53 and 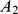 to be the amplitude of the second peak. Sensitivity to the second pulse is defined as the difference between 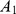 and 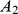, with 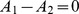 indicating full sensitivity. As seen in Figures 4D and E, the flux of p53 has no bearing on the sensitivity to the second pulse while the flux of Mdm2 strongly affects it. At one one-hundredth the observed Mdm2 flux – corresponding to protein half-life of 3-days – over 20,000 fewer molecules of p53 are phosphorylated, representing more than a two-fold reduction in sensitivity (Figure 4E). This result is robust with respect to the interval of time chosen between pulses (Figure S2). If the sensitivity to the second pulse is already compromised by a reduced Mdm2 flux, a concomitant reduction in p53 flux fails to rescue it, while an increase in p53 flux still further reduces it (Figure 4F). We therefore conclude that the flux of Mdm2, and not p53, controls the system's refractory time, and a high Mdm2 flux is required to re-establish sensitivity after transient stimulation.

### High IκB flux buffers NFκB from activation in response to UV and low doses of TNF

A second major stress-response pathway is that of NFκB. NFκB is potently induced by the inflammatory cytokine TNF, but shows a remarkable resistance to internal metabolic perturbations or ribotoxic stresses induced by ultraviolet light (UV) [13], or to triggers of the unfolded protein response (UPR) [28]. Like p53, the dynamics of NFκB activation play a major role in determining target gene expression programs [29], [30]. Although NFκB is considered stable, the flux of IκBα – the major feedback regulator of NFκB – is conspicuously high. We hypothesized that turnover of IκB controls the stimulus-responsiveness of the NFκB signaling module.

**Figure 5:**
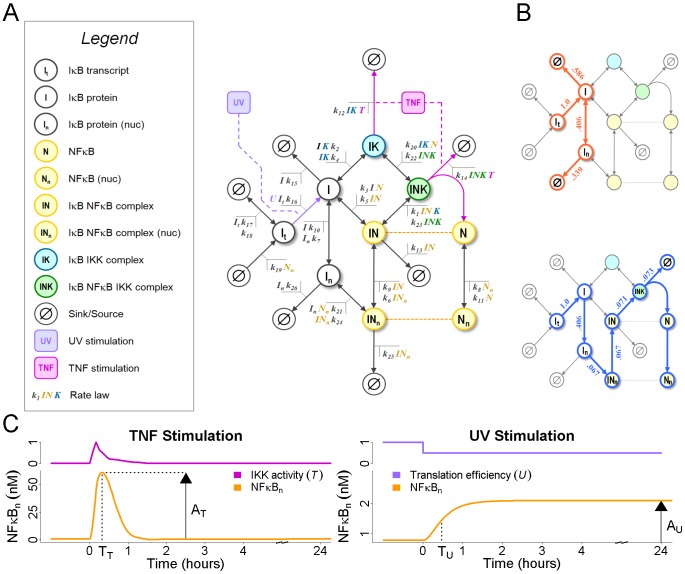
A model of IκB-regulated NFκB activation. (A) An IκB-centric diagram of NFκB regulation. IκB is transcribed in an NFκB-dependent and -independent manner. Translated IκB may bind to IKK (cyan), NFκB (yellow), or both (green), or it may shuttle to the nucleus and bind to NFκB there. Degradation of IκB is possible from any state, though only when bound to IKK can degradation be enhanced by IKK activity. Activation of NFκB is achieved by the time-dependent numerical inputs 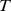 (magenta) and 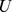 (violet). 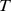 represents the activity of IKK kinase while 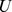 is the efficiency of mRNA translation. Both are defined over the interval 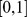, with 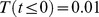 and 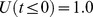 being their wildtype, unstimulated values. (B) The futile (red) and productive (blue) IκB degradation fluxes. The fraction of total IκB flux through each reaction is listed next to the corresponding reaction arrow. (C) Two stimuli used in our analysis of NFκB activation and the effects of IκB flux. Stimulation by TNF is modeled using the time-dependent IKK activation profile described in [29] and results in strong but transient activation of NFκB. Stimulation by UV is modeled as a 50% reduction of translational efficiency, as described in [13], and results in modest but sustained activation. As with p53, we define 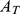 and 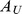 to be the maximum activity of NFκB in response to TNF and UV, respectfully, and 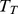 to be the time at which 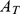 is observed. Because 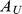 is observed infinitely often, we define 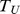 to be the time at which NFκB activation reaches one-half 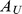.

Beginning with a published model of NFκB activation [13], we removed the beta and epsilon isoforms of IκB, leaving only the predominant isoform, IκBα (hereafter, simply “IκB”; Figure 5A). Steady state analysis of this model supported the observation that almost all IκB is degraded by either of two pathways: a “futile” flux, in which IκB is synthesized and degraded as an unbound monomer; and a “productive” flux, in which free IκB enters the nucleus and binds to NFκB, shuttles to the cytoplasm, then binds to and is targeted for degradation by IKK (Figure 5B). These two pathways account for 92.5% and 7.3% of the total IκB flux, respectively. The inflammatory stimulus TNF was modeled as before, using a numerically-defined IKK activity profile derived from *in vitro* kinase assays [30] (Figure 5A, variable 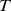). Stimulating with TNF results in strong but transient activation of NFκB. A second stimulus, ribotoxic stress induced by UV irradiation, was modeled as 50% reduction in translation and results in only modest activity [13]. As above, we let 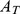 be the amplitude of activated NFκB in response to TNF and 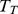 the time at which 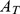 is observed. Analogously, we let 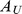 be the amplitude of NFκB in response to UV, and 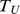 the time at which NFκB activation equals one-half 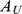 (see Figure 5C). We then implemented multipliers for the futile and productive flux and let each multiplier take values on the interval 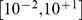. For each value we simulated the NFκB response to TNF and UV and plotted the effects on 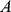 and 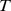.

**Figure 6:**
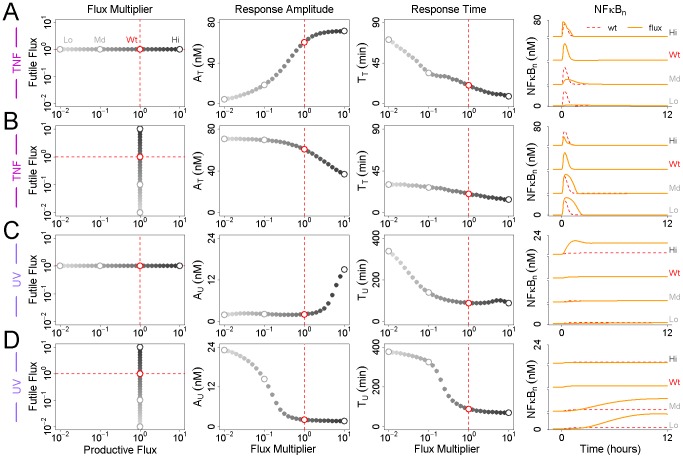
Effects of IκB flux on the NFκB response to stimulation. (A) The productive flux of IκB was varied between 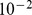 and 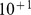 times its wildtype value prior to stimulation by TNF (light gray to dark gray), and the resulting NFκB response values 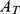 and 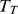 plotted in columns 2 and 3. Representative nuclear NFκB profiles for low, moderate, wildtype, and high values of the flux multiplier are shown at right. Again, the wildtype productive flux is indicated by the dashed line in red. (B) The futile flux of IκB was varied between 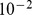 and 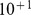 times its wildtype value prior to stimulation by TNF and the resulting NFκB response values 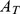 and 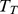 plotted in columns 2 and 3. (C) and (D) As rows 1 and 2, above, but the response to UV stimulation is plotted instead of TNF.

The results show that reducing the productive flux yields a slower, weaker response to TNF (Figure 6A). By analogy to Figure 2, this indicates that the productive flux of IκB is a positive regulator of NFκB activation. In contrast, the futile flux acts as a negative regulator of NFκB activity, though its effects on 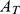 and 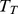 are more modest (Figure 6B). Thus, similar to p53, the activation of NFκB is controlled by a positive and negative regulatory flux. In response to UV, a reduction in either flux delays NFκB activation, but reducing the futile flux results in a significant increase in 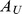 while reducing the productive flux has almost no effect (Figure 6C and D). Conversely, while an increase in the futile flux has no effect on 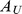, an increase in the productive flux results in a significant increase. If we now define NFκB to be sensitive to TNF or UV when 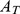 or 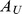 are ten-fold higher than its active but pre-stimulated steady state abundance, then TNF sensitivity requires a productive flux multiplier 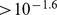, while UV insensitivity requires a productive flux multiplier 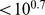 and a futile flux multiplier 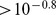. This suggests that the flux pathways of IκB may be optimized to preserve NFκB sensitivity to external inflammatory stimuli while minimizing sensitivity to internal metabolic stresses.

**Figure 7:**
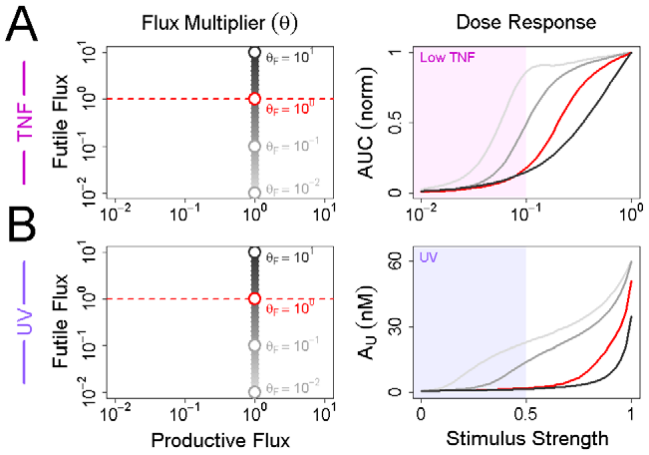
IκB flux controls the sensitivity of NFκB to stimulation by TNF and UV. (A) The futile flux of IκB was varied between 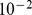 and 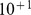 times its wildtype value prior to stimulation with variable doses of TNF (see Methods). For low, medium, high, and wildtype values of the futile flux, the area under the NFκB activation curve is plotted as a function of TNF dose. The region of the plot corresponding to low doses of TNF, where the activation of IKK does not exceed 10%, is shaded in pink. (B) As above, but variable doses of UV are used instead of TNF. Because the response to UV is sustained and not transient, we have plotted the value of 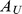 as a function of UV dose instead of the area under the NFκB activation curve.

In contrast to p53, the negative regulatory flux of IκB dominates the positive flux. We hypothesized that this imbalance must affect the sensitivity of NFκB to weak stimuli. To test this hypothesis we generated dose-response curves for TNF and UV using the following multipliers for the futile flux: 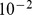, 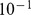, 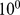, and 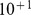 (see Methods). The results confirm that reducing the futile flux of IκB results in hypersensitivity at low doses of TNF (Figure 7, Row 1). At one one-hundredth the wildtype flux, a ten-fold weaker TNF stimulus yields an equivalent NFκB response to the full TNF stimulus at the wildtype flux. Similarly, a high futile flux prevents strong activation of NFκB in response to UV (Figure 7, Row 2). At 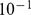 and 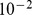 times the futile flux, UV stimulation results in a 20-fold increase in NFκB activity, compared to just a 2-fold increase at the wildtype flux. We therefore conclude that turnover of unbound IκB controls the EC50 of the NFκB signaling module, and that rapid turnover renders NFκB resistant to metabolic and spurious inflammatory stimuli.

## Discussion

Previous studies have shown that the fluxes of p53 [10], [11], its inhibitor Mdm2 [31], [32], and the unbound negative regulator of NFκB, IκB [12], are remarkably high. To investigate whether rapid turnover of these proteins is required for the stimulus-response behavior of the p53 and NFκB stress response pathways, we developed a computational method to alter protein turnover, or flux, independently of steady state protein abundance.

For p53, we show that high flux is required for sensitivity to sustained stimulation after ionizing radiation (Figure 4A). Interestingly, inactivating mutations in p53 have long been known to enhance its stability [33], either by interfering with Mdm2-catalyzed p53 ubiquitination [34], [35], or by affecting p53's ability to bind DNA and induce the expression of new Mdm2 [36]–[39]. Inactivation of p53 also compromises the cell's sensitivity to IR [40], [41]–[43]. Our results offer an intriguing explanation for this phenomenon, that p53 instability is required for oscillations in response to IR. Indeed, IR sensitivity was shown to correlate with p53 mRNA abundance [44]–[46], a likely determinant of p53 protein flux. In further support of this hypothesis, mouse embryonic fibroblasts lacking the insulin-like growth factor 1 receptor (IGF-1R) exhibit reduced p53 synthesis and degradation, but normal protein abundance. These cells were also shown to be insensitive to DNA damage, caused by the chemotherapeutic agent etoposide [32].

Like p53, increased stability of Mdm2 has been observed in human leukemic cell lines [47], and Mdm2 is a strong determinant of IR sensitivity [48], [49]. Again our results suggest these observations may be related. Activation of p53 in response to IR is mediated by the ATM kinase (“Signal” in Figure 3) [50], [51]. Batchelor *et al*. show that saturating doses of IR result in feedback-driven pulses of ATM, and therefore p53 [17]. In Figure 4B we show that these are independent of Mdm2 flux. However, sub-saturating doses of IR (10 Gy versus 0.5 Gy) [52], [53] cause only transient activation of ATM [54], after which constitutive Mdm2 synthesis is required to restore p53 sensitivity (Figure 4E). This suggests that high Mdm2 flux is required for sensitivity to prolonged exposure to sub-saturating doses of IR. Indeed, this inverse relationship between flux and refractory time has been observed before. In Ba/F3 pro-B cells, high turnover of the Epo receptor maintains a linear, non-refractory response over a broad range of ligand concentrations [55].

For NFκB, our method revealed that an isostatic reduction in the half-life of IκB sensitizes NFκB to TNF (Figure 7A), as well as to ribotoxic stress agents like UV (Figure 7B). This observation agrees with previous theoretical studies using a dual kinase motif, where differential stability in the effector isoforms can modulate the dynamic range of the response [56]. For NFκB, the flux of free IκB acts as a kinetic buffer against weak or spurious stimuli, similar to serial post-translational modifications on the T cell receptor [57], or complementary kinase-phosphatase activities in bacterial two-component systems [58]. In contrast, increasing the half-life of IκBα alone – without a coordinated increase in its rate of synthesis – increases the abundance of free IκBα and actually dampens the activity of NFκB in response to TNF [25]. This difference highlights the distinction between isostatic perturbations and traditional, unbalanced perturbations that also affect the steady state abundances. It also calls attention to a potential hazard when trying to correlate stimulus-responsiveness with protein abundance measurements: observed associations between responses and protein abundances do not rule out implied changes in kinetic parameters as the causal link. Indeed static, and not kinetic measurements, are the current basis for molecular diagnosis of clinical specimens. Thus while nuclear expression of p53 [59]–[66] and NFκB [67]–[69] have been shown to correlate with resistance to treatment in human cancer, the correlation is not infallible [40], [70]–[74]. If stimulus-responsiveness can be controlled by protein turnover independently of changes to steady state abundance, then correlations between abundance and a therapeutic response may be masked by isostatic heterogeneity between cells.

**Figure 8:**
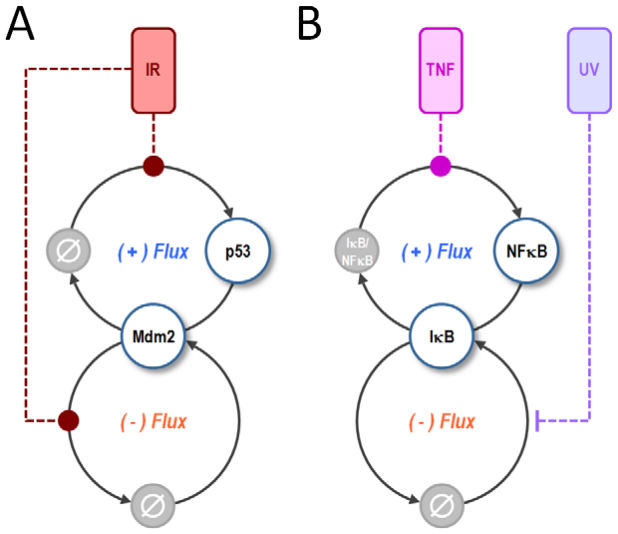
A paired positive (+) and negative (−) flux motif controls stimulus-sensitivity in the p53 and NFkB stress response pathways. (**A**) For p53, the (+) flux is formed by the synthesis and degradation of p53 itself. The (−) flux is formed by synthesis and degradation of Mdm2. Together these fluxes control the sensitivity of p53 to IR-stimulation, which acts by inducing the synthesis of p53 and the degradation of Mdm2. (B) For NFkB, the (+) flux is formed by association and dissociation of NFkB from its negative regulator, IκB. The (−) flux is formed by synthesis and degradation of IκB. These fluxes control the sensitivity of NFkB to TNF-stimulation, which induces the dissociation of NFκB from IκB, and UV-stimulation, which inhibits the synthesis of IκB.

For p53 and NFkB, we show that stimulus sensitivity can be controlled by a paired positive and negative regulatory flux. We propose that this pairing may constitute a common regulatory motif in cell signaling. In contrast to other regulatory motifs [75], [76], the “flux motif” described here does not have a unique structure. The positive p53 flux, for example, is formed by the synthesis and degradation of p53 itself, while the positive flux in the NFκB system includes the nuclear import of free NFκB and export of NFκB bound to IκB. For p53, the negative flux is formed by synthesis and degradation of Mdm2, while for NFκB it is formed by the synthesis, shuttling, and degradation of cytoplasmic and nuclear IκB. Thus the reaction structure for each flux is quite different, but they nevertheless form a regulatory motif that is common to both pathways (Figure 8). And since the mathematical models used here are only abstractions of the underlying network, the true structure of the p53 and NFκB flux motifs are in reality even more complex.

The identification of a flux motif that controls stimulus-responsiveness independently of protein abundances may prompt experimental investigation into the role of flux in signaling. At a minimum, this could be achieved using fluorescently-labeled activator and inhibitor proteins in conjunction with tunable synthesis and degradation mechanisms. The tet-responsive promoter system [77], [78], for example, could provide tunable synthesis, while the CLP-XP system [79] could provide tunable degradation. For the two-dimensional analysis presented here, and to avoid confounding effects on signaling dynamics caused by shared synthesis and degradation machinery [80], independently tunable synthesis and degradation mechanisms may be required. If these techniques are applied to mutants lacking the endogenous regulators, this would further allow decreases in protein flux to be studied in addition to strictly increases.

Finally, in this study we have examined the effects of flux on stimulus-responsiveness, but in a typical signaling module, many other isostatic perturbations exist. For example, the isostatic subspace of our NFκB model has 18 dimensions, of which only a few were required by the analysis presented here. By simultaneously considering all isostatic perturbations, some measure of the dynamic plasticity of a system can be estimated, perhaps as a function of its steady-state. Such an investigation can inform diagnosis of biological samples, and whether information from a single, static observation is sufficient to predict the response to a particular chemical treatment, or whether live-cell measurements are required as well. As we have shown that protein turnover can be a powerful determinant of stimulus-sensitivity, we anticipate that kinetic measurements will be useful predictors of sensitivity to chemical therapeutics.

## Methods

### Modeling isostatic perturbations in protein turnover

To begin, we assume that the system of interest has been modeled using mass action kinetics and that the steady state abundance of every biochemical species is a known function of input parameters. In other words,
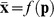
such that
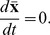
(1)


Equation 1 is the well-known steady-state equation; 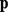 is a vector of independent parameters and 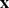 is the vector of species abundances. We use an overbar to denote a vector 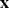 that satisfies Equation 1. For excellent reviews on mass action models and their limitations, see [81]–[83]. For a method on finding analytical solutions to the steady state equation, see our accompanying manuscript. Next, we wish to find a change 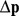 in the input parameters such that the resulting change 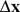 in the species abundances is zero, where 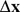 is defined as
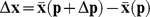



Thus for 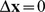, we require that
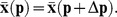



The right-hand side of this equation can be approximated by a truncated Taylor series, as follows:
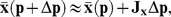
where 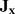 is the Jacobian matrix whose elements are the partial derivatives of each species with respect to each parameter. Thus, for 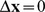 we require that
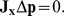



In other words, 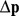 must lie in the null space of 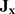. We call this the *isostatic subspace* of the model – parameter perturbations in this subspace will not affect any of the steady-state species abundances. If 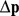 lies within the isostatic subspace, it is an *isostatic perturbation vector*. Let 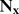 be a matrix whose columns form a basis for the isostatic subspace. Then a general expression for an isostatic perturbation vector is simply
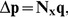
(2)where 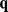 is a vector of unknown basis vector coefficients. Finally, Equation 2 can be solved for a specific linear combination of basis vectors that achieves the desired perturbation. In our case we identified those combinations that result in changes to protein turnover.

### A prototypical negative feedback model

Our prototypical negative feedback model consists of two species, an activator “X” and an inhibitor “Y”, and four reactions, illustrated in Figure 1A. Let 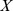 denote the abundance of the activator and 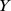 denote the abundance of the inhibitor. An analytical expression for the steady-state of this model was identified by solving Equation 1 for the rates of synthesis, giving
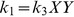
(3)

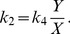
(4)


To parameterize the model we first let 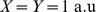. Degradation rate constants were then calculated such that 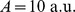 at time 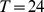, where again 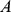 is the maximum amplitude of the response. Activation was achieved by instantaneous reduction of 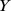 to 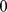. To modify the flux, we defined flux multipliers 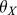 and 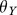 such that 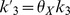 and 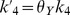. Note that by virtue of Equations 3 and 4, values for 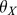 and 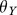 other than 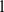 result in commensurate changes in 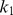 and 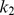 such that steady state is preserved. See file “pnfm.sci” in Protocol S1 for details. Figures 2A and 2B were achieved by letting 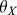 and 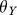 vary over the interval 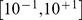, then calculating the altered vector of rate constants 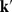 and simulating the model's response to stimulation. Figure 2C required letting 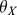 vary over this same interval while having 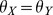. Finally, Figure 2D was achieved by letting 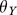 vary over the same interval, and for each value of 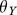, numerically calculating the value of 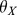 that gave 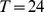.

### A model of p53 oscillations

All species, reactions, and rate equations required by our model of p53 oscillations are as previously described [17]. Our only modification was to scale the parameter values so that the rates of p53 and Mdm2 synthesis and degradation, as well as their steady-state abundances, matched published observations (see Table S1). Specifically we let
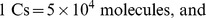


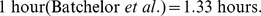



To derive a steady-state solution for this model, we solved Equation 1 for the steady-state abundance of Mdm2 and the rate of Mdm2-independent p53 degradation, giving
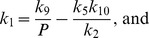


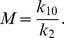



To simulate the response to ionizing radiation we used the (scaled) stimulus given in [17]. Namely, at time 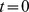 we let the rate of Signal production, 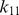, go to 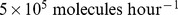. This stimulus was either maintained indefinitely (Figure 4A–C) or for just 2 hours, followed by 22 hours of rest, followed by a second 2 hour stimulation (Figure 4D–F). Changes in p53 or Mdm2 flux were achieved as above, by defining modifiers 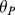 and 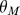 such that
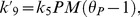
(5)

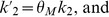
(6)

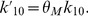
(7)


Prior to stimulation, we let one modifier take values on the interval 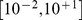 while holding the other modifier constant. Equations 6 and 7 ensure that the p53-independent flux of Mdm2 is modified without affecting its steady-state abundance. Equation 5, which is slightly more complicated, results in changes to the rate of Mdm2-independent p53 degradation, 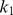, by modifying the independent parameter 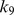, which controls the rate of p53 synthesis. This yields the desired
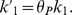



Numerical integration was carried out to time 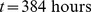. After each integration, we defined 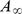 to be the minimum vertical distance between any adjacent peak and trough in phosphorylated p53, and 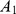 and 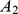 to be the amplitudes of the first and second peak, respectively. Details of this model can be found in the file “p53b.sci” in Protocol S1. For more information on the time delay parameters 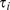 and 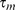, and their role in generating oscillations, see [84], [85].

### A model of NFκB activation

Our model of NFκB activation is similar to the one described in [13], except the beta and epsilon isoforms of IκB have been removed. Our model has 10 species and 26 reactions, the majority of which are illustrated in Figure 5A. Rate equations and parameter values are identical to those in [13]. An analytical expression for the steady-state of this model was found by solving Equation 1 for the following dependent variables: 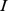, 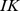, 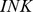, 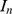, and 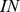, and the rate constants 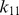, 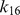, and 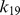. The precise expressions for these variables are extremely cumbersome but may be found in their entirety in the file “nfkb.sci” in Protocol S1.

Activation of NFκB is achieved by either of two, time-dependent numerical input variables, 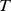 and 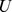. 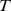 modifies the activity of IKK while 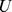 modifies the efficiency of IκB translation. Both have a finite range of 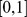 and have unstimulated, wildtype values of 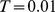 and 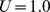, respectively. The inflammatory stimulus TNF is modeled using a unique function of 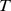 derived from *in vitro* kinase assays [30]. Since these assays only measured IKK activity out to 4 hours, we extended each stimulus by assuming the value of 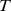 at 4 hours is maintained out to 24 hours. Justification for this can be found in the 24-hour kinase assays in [86], which shows no IKK activity between 8 and 24 hours after TNF stimulation. UV stimulation is modeled using a step decrease in the value of 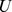 from 1.0 to 0.5 for the entire 24 hours. This mimics the 50% reduction in translational efficiency observed in [13].

Steady-state analysis of this model revealed that over 99% of all IκB was degraded via either of two pathways, futile (92%) and productive (7%). See Figure 5B for the composition of these pathways. To modify the flux through either pathway without altering any of the steady-state abundances, the algebraic method described above proved absolutely necessary. Specifically, we solved Equation 2 for the unique set of basis vector coefficients such that the following conditions held: (1) only reaction rate constants involved in the targeted pathway were modified; (2) if a reaction on the pathway was reversible, its ratio of forward to reverse rate constants was preserved; and (3) the magnitude of an alteration was relative to the bottleneck reaction. For the futile flux this was 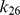, the degradation of unbound nuclear IκB. For the productive flux it was 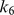, the export of NFκB-bound IκB. As in the p53 models above, we then defined multipliers 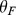 and 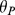 such that
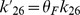


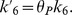



See file “nfkb.sci” in Protocol S1 for the precise effect of 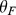 and 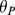 on the other rate constants in the model. Finally, to generate Figure 6 we let the appropriate multiplier take values on the interval 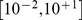 prior to stimulation with TNF or UV.

Dose response curves in Figure 7 were generated by letting 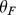 take values in 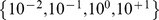 and simulating the response to varying doses of TNF or UV. To vary the TNF dose, we scaled the displacement of the numerical IKK activation curve above its basal value of 1% using log-spaced multipliers on the interval 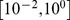. We call this multiplier the “stimulus strength”. A stimulus strength of 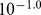, for example, yields the same basal IKK activity as the full TNF dose used in Figure 6, but a peak activity whose magnitude is just one-tenth that of the full dose. To measure the TNF response, we calculated an area under the curve (AUC) by subtracting NFκB basal activity from the TNF-induced NFκB activation curve, then integrated this curve from the point of stimulus to the time at which it becomes less than one-tenth the basal activity. All AUCs were normalized to the full TNF dose. To vary the UV dose we varied the magnitude of the displacement of 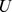 from unity. A stimulus strength of 0.1, for example, results in a step decrease in 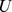 from 1.0 to 0.9. Because the response to UV is sustained instead of transient, we plotted 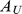 as a function of stimulus strength instead of the area under the curve.

## Supporting Information

Figure S1
**Locations of Mdm2, p53, and IκBα in a genome-wide distribution of protein flux.** A histogram of protein flux was generated from data in [4] 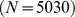. Assuming first-order degradation kinetics, the published half-life for each protein was used in conjunction with its steady-state abundance to calculate its rate of synthesis. This rate was then divided by the steady-state abundance to derive each protein's *normalized flux*, that is, the fraction of its steady-state population that is synthesized every hour. Normalized flux values for Mdm2, p53, and unbound IκBα are indicated by the dashed lines. Daggers denote proteins whose half-lives are extrinsic to the dataset.(TIF)

Figure S2 **Choice of interval time does not affect the role of Mdm2 flux in p53 refractory time.** This plot is identical to Figure 4D–E, except that the interval between pulses is taken to be 6 (magenta), 12 (yellow), 24 (green), or 48 hours (cyan). Representative traces at right are grouped according to interval time.
(TIFF)

Protocol S1 **Executable source code.** This zip file contains executable code for all three models discussed in the manuscript. All code can be executed using the freely available Scilab numerical computing environment, http://www.scilab.org/. See the included README file for detailed instructions.
(ZIP)

Table S1 **Parameter values used to simulate the model of p53 oscillations.** This table lists all parameters required to simulate the model of p53 oscillations. Parameters with published values are shown for comparison.
(DOCX)
