# Supplementary figures and images for: A Protein Turnover Signaling Motif Controls the Stimulus-Sensitivity of Stress Response Pathways
Source: PLoS Comput Biol. 2013 Feb 28;9(2):e1002932. doi: 10.1371/journal.pcbi.1002932 (PMC3585401; doi:10.1371/journal.pcbi.1002932)

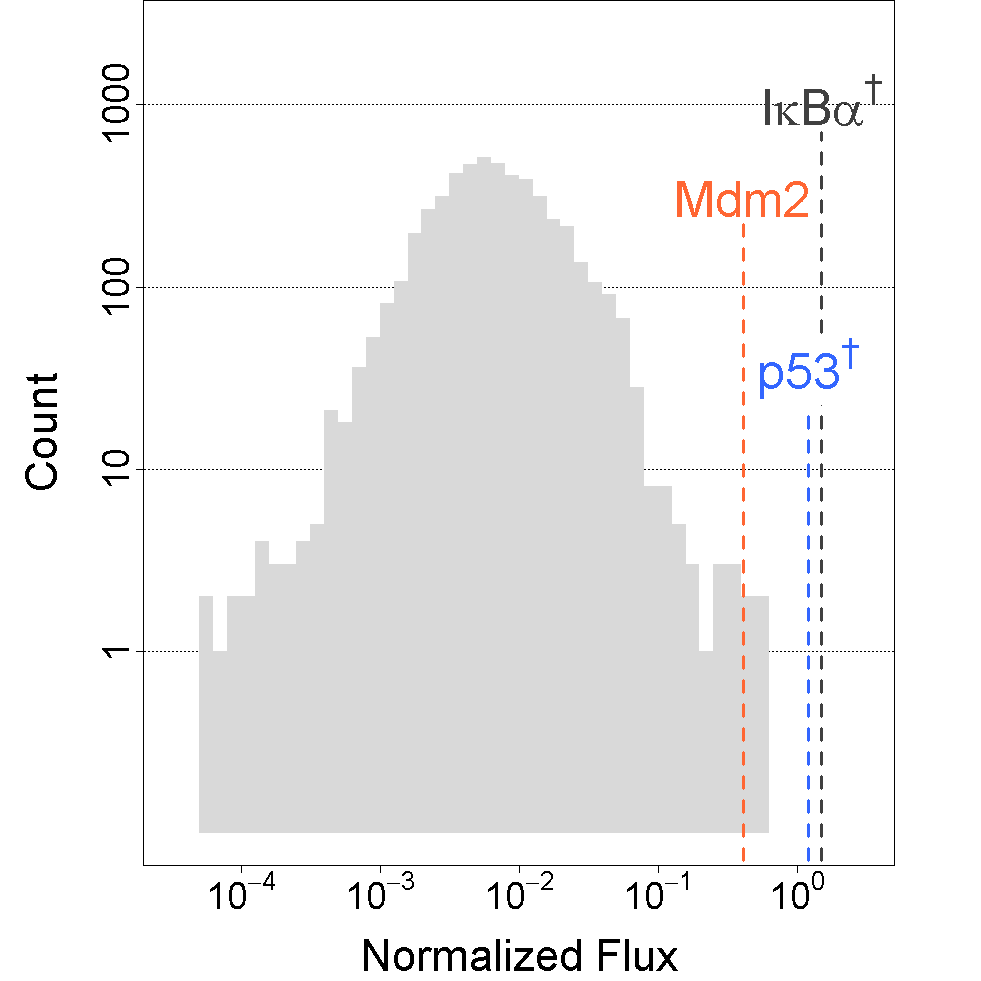

Supplement: Figure S1 — Locations of Mdm2, p53, and IκBα in a genome-wide distribution of protein flux. A histogram of protein flux was generated from data in [4] . Assuming first-order degradation kinetics, the published half-life for each protein was used in conjunction with its steady-state abundance to calculate its rate of synthesis. This rate was then divided by the steady-state abundance to derive each protein's normalized flux, that is, the fraction of its steady-state population that is synthesized every hour. Normalized flux values for Mdm2, p53, and unbound IκBα are indicated by the dashed lines. Daggers denote proteins whose half-lives are extrinsic to the dataset. (TIF) [file pcbi.1002932.s001.tif]

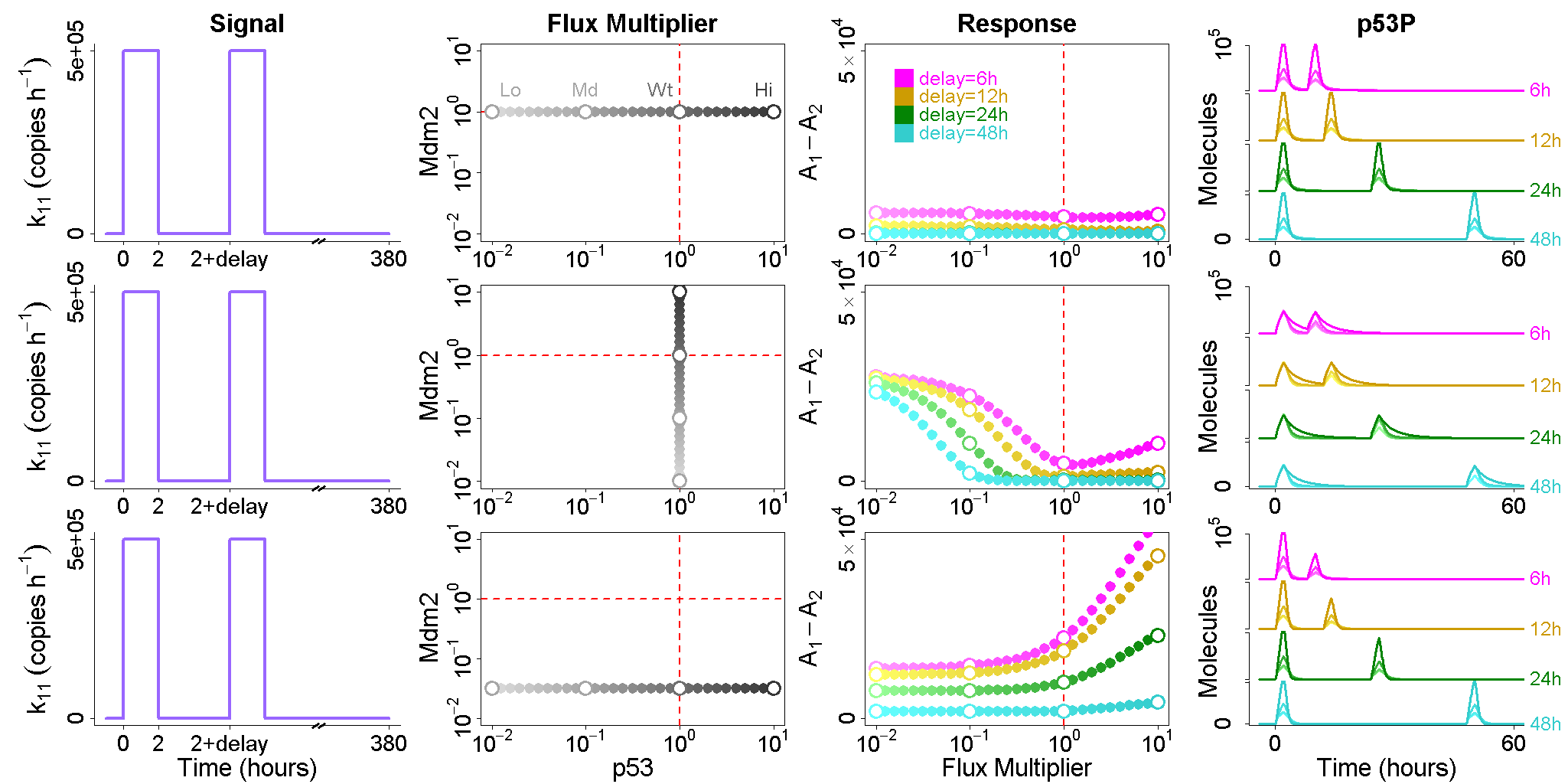

Supplement: Figure S2 — Choice of interval time does not affect the role of Mdm2 flux in p53 refractory time. This plot is identical to Figure 4D–E, except that the interval between pulses is taken to be 6 (magenta), 12 (yellow), 24 (green), or 48 hours (cyan). Representative traces at right are grouped according to interval time. (TIFF) [file pcbi.1002932.s002.tiff]
