# Supplementary material for: A Protein Turnover Signaling Motif Controls the Stimulus-Sensitivity of Stress Response Pathways
Source: PLoS Comput Biol. 2013 Feb 28;9(2):e1002932. doi: 10.1371/journal.pcbi.1002932 (PMC3585401; doi:10.1371/journal.pcbi.1002932)
Supplement: Table S1 — Parameter values used to simulate the model of p53 oscillations. This table lists all parameters required to simulate the model of p53 oscillations. Parameters with published values are shown for comparison. (DOCX) [file pcbi.1002932.s004.docx]

Table S1. Parameters used in a model of p53 oscillations.

| Param | Meaning | Value | Units^[[1]](#footnote-1)^ | Pub^[[2]](#footnote-2)^ | Ref^[[3]](#footnote-3)^ |
| --- | --- | --- | --- | --- | --- |
| *x*_1_ | Initial p53 inactive concentration | 1.5e4 | Cs | 2.0e4 | [Ma] |
| *x*_3_ | Initial Mdm2 concentration | 1e4 | Cs | 1e4 | [4] |
| *k*_1_ | Inactive p53 degradation rate | 1.5 | h^-1^ | 1.18 | [10] |
| *k*_2_ | Mdm2 degradation rate | 0.75 | h^-1^ | 0.92 | [4] |
| *k*_3_ | Inhibitor degradation rate | 0.525 | h^-1^ |  |  |
| *k*_4_ | Inhibitor-independent Signal degradation rate | 5.63 | h^-1^ |  |  |
| *k*_5_ | Mdm2-dependent p53inactive degradation rate | 7.5e-5 | Cs^-1^ h^-1^ |  |  |
| *k*_6_ | Mdm2-dependent p53active degradation rate | 2.1e-5 | Cs^-1^ h^-1^ |  |  |
| *k*_7_ | Signal-dependent Mdm2 inactivation rate | 7.5e-6 | Cs^-1^ h^-1^ |  |  |
| *k*_8_ | Saturating Inhibitor-dependent Signal degradation rate | 37.5 | h^-1^ |  |  |
| *k*_9_ | p53 inactive production rate | 3.38e4 | Cs h^-1^ | 2.4e4 | [10]^[[4]](#footnote-4)^ |
| *k*_10_ | p53-independent Mdm2 production rate | 7.5e3 | Cs h^-1^ | 4.2e3 | [4] |
| *k*_12_ | p53-dependent Mdm2 production rate | 0.675 | h^-1^ |  |  |
| *k*_13_ | Inhibitor production rate | 0.15 | h^-1^ |  |  |
| *k*_14_ | Saturating production rate of p53active | 7.5 | h^-1^ |  |  |
| *c*_1_ | Hill coefficient of Signal degradation by Inhibitor | 4.0 | unitless |  |  |
| *c*_2_ | Hill coefficient of active p53 production by Signal | 4.0 | unitless |  |  |
| *c*_3_ | Inhibitor concentration for half-maximal Signal degradation | 1.0e4 | Cs |  |  |
| *c*_4_ | Signal concentration for half-maximal p53 production | 5.0e4 | Cs |  |  |
| *c*_5_ | Time delay in Mdm2 production | 0.933 | h |  |  |
| *c*_6_ | Time delay in Inhibitor production | 1.6 | h |  |  |

1. 1 Cs is equivalent to 1 copy of the indicated molecule. 1 h is 1 hour. [↑](#footnote-ref-1)
2. Published value. Half-lives converted to rate constants by inverting and multiplying by ln(2). [↑](#footnote-ref-2)
3. Citation for published value. Citation number matches that in main text. [↑](#footnote-ref-3)
4. Value calculated by multiplying p53 steady state abundance (2e4) by degradation rate constant (1.18). [↑](#footnote-ref-4)
